# Exploring biophilic building designs to promote wellbeing and stimulate inspiration

**DOI:** 10.1371/journal.pone.0317372

**Published:** 2025-03-04

**Authors:** Yangang Xing, Nikki Stevenson, Carolyn Thomas, Alex Hardy, Andrew Knight, Nadja Heym, Alex Sumich

**Affiliations:** 1 School of Architecture, Design and Built Environment, Nottingham Trent University, Nottingham, England; 2 School of Social Science, Psychology, Nottingham Trent University, Nottingham, England; King Khalid University, EGYPT

## Abstract

*Biophilic designs* aim to promote health and wellbeing by incorporating nature-based features into internal and external built environments. Three theories have previously been proposed (i.e., *Recovery, Attention Restoration, Refuge, and Prospect*) regarding the impact of biophilic features on psychological and physiological health, but with little empirical evaluation. This current study tests these three existing theories, alongside a novel biophilic theory proposed in this paper, as that biophilic environments stimulate *inspiration*. A public survey was conducted, and participants completed an online stress-induction task followed by images of building interiors that systematically varied in perceived biophilic quality—ranging across four levels (from 0 = no clear biophilic features to 3 = very high biophilic features). Participants rated their psychological states associated with each of the proposed theories before and after each trial’s stress-induction and biophilic phases. Results support a positive effect of exposure to biophilic design on self-reported psychological states (including inspiration), whilst designs without biophilic quality tended to have an adverse effect on psychological states. Furthermore, findings support the extension of the current three theories to include the impact of biophilic designs on stimulating inspiration.

## 1. Introduction

The term “Biophilia”—meaning love of life—was first mentioned by the psychoanalyst, Erich Fromm, who observed that our separation from nature through industrialisation adversely impacts mental health [1]. Biophilia theory posits an innate need to affiliate with nature to derive aesthetic, physiological, emotional, cognitive, and spiritual resources [2]. The biophilia theory lately extends to biophilic building designs to promote a connection between people and nature through the incorporation of natural design features [3–5]. The notion that biophilic design features may positively impact psychological states, aligns with the critical needs to promote healthier buildings and healthier living in the context of rapid urbanisation on a global scale [6,7]. Currently, over 55% of the world’s population lives in urban areas – a proportion that is expected to increase to 68% by 2050 [8]. City dwellers typically spend around 90% of their time in urban environments [9]. [10]Compared to living in and around green spaces, urban living is associated with poorer physical [11], and mental health and stress [12]. Furthermore, in the UK, 25% of office workers believe that their working environment does not support their wellbeing, with particular problems including the absence of colour (80%), greenery (64%), and art (61%) in their workplaces [13]. The Human Spaces Survey reports that 58% of participants did not have any plants in their offices and 47% of 7,600 office workers (spanning 16 countries) felt stressed in their workplace [14]. These highlight the health risks linked to living environments that lack biophilic elements [10].

[2,15,16]Whilst traditional psychological perspectives focus on a deficit model, aiming to improve some condition or symptoms (e.g., anxiety), a growing area of interest involves improving the lives of people not suffering from a recognised mental health condition but attempting to live more fulfilled and flourishing lives. Seligman is credited with the renaissance of modern psychology studies examining human flourishing. His term, *positive psychology*, is defined as a study of positive experiences, positive character traits, and the situations that help cultivate them [17]. Studies have demonstrated that participants could undertake Positive Psychology Exercises (PPE) to improve their happiness and wellbeing [17] Thus, the aim of the current study, partly influenced by the positive psychology movement, was to examine to what extent biophilic environments can increase the wellbeing of the general population, rather than those suffering from a particular mental health problem. There is currently a design revolution with a drive to incorporate more natural and living materials (e.g., wooden features, plants, and green walls) into our urban environments to increase positive psychological wellbeing [2,15,16].

Current theories regarding the positive benefits of biophilic design features primarily focus on three main concepts [5,18,19]: *Restoration of cognitive resources* [20]; *Stress reduction and recovery* [21]; and provision of *Refuge and prospect* [22]. An important aspect of human wellbeing, not covered by the three existing theories is that of *inspiration*. Thus, in addition to the three established theories, we proposed a fourth theory, namely that biophilic environments stimulate *creativity* and *Inspiration*. The idea that inspiration is somehow generated external to the individual dates back to classical antiquity. Pappas [23] notes that both Homer and Hesiod begin their works by asking a muse to speak to them. Throughout Plato’s writings, there are references to the muses who act as divine inspiration for poets. Hence, the notion of being taken over by some form of inspiration has a long and rich tradition [23]. Nevertheless, in a landmark study, Thrash and Elliot [24] recognise that inspiration has received little theoretical or empirical attention within psychology. They proceeded to undertake a conceptualisation of the term *inspiration* and argue that inspiration is evoked, rather than initiated directly by an active will, and involves “transcendence of the ordinary preoccupation or limits of human agency” (p. 871). Thrash and Elliot [24] continue with a theme that reaches back to Plato but bring increased conceptual clarity to the problem. They usefully distinguish between triggers for inspiration and the targets towards which inspiration is directed. So, for instance, a feeling of inspiration may result from sitting in a quiet woodland glade on a summer evening. This may manifest itself in a range of targets, for illustration, the inspiration to undertake a creative activity such as painting. It is, therefore, clear that inspiration is a valuable and positive feeling that enhances our lives. Therefore, one purpose of the current study was to test if a biophilia setting can increase feelings of inspiration, as self-described by participants.

Stimulating Inspiration is important in improving cognitive function and enhancing creativity, leading to improved productivity [3,14]. However, inspirational and other mental health aspects of buildings are often overlooked for several reasons [5]. Primarily, owing to a focus on function over aesthetics; building design is often driven solely by over-simplified functional considerations (e.g., thermal comfort), which can lead to uninspiring buildings that lack visual appeal [15]. Building projects are often limited by available funding and cost control, resulting in a focus on necessities rather than inspiring design elements. The focus on stet necessities can result in a neglect of potential long-term benefits of an inspiring building. For example, the homogeneity of buildings in many cities, characterised by buildings that are similar in appearance (internally and externally) and lack uniqueness, makes it difficult for city dwellers to attain mental health benefits [8]. Critically, there is a need to explore design principles and practices that can contribute to an inspiring building since this has not been adequately researched [8,25,26]. Establishing a greater understanding of these benefits is essential to developing more holistic theories and can help inform decisions on various building policies, designs, and expenditures [6]. Thus, alongside the development of green building rating schemes to assess building sustainability and its potential impacts on wellbeing, a systematic assessment of the effect of biophilic environments on psychological and physiological states, and consequently wellbeing in the context of these theoretical frameworks is needed.

Taken together, the key research question explored in this paper is to what extent varied levels of biophilic design impact emotional states as mapped on to four biophilic theories, namely *Recovery, Attention Restoration, Refuge and Prospect,* and *Inspiration*. As biophilic design is a complex phenomenon involving multiple features (e.g., natural organisms, but also abstract forms of nature), different elements of biophilic quality were incorporated into virtually designed spaces. Moreover, this study focused on the role of biophilic environments in educational spaces for several reasons: i) biophilic research in education and workplace is in its infancy - compared to occupational environments, relatively little previous research has examined the beneficial impact of biophilic features in educational environments [27]; ii) educational environments pose a challenge to meet a range of different, sometimes clashing, individual needs across wellbeing, learning and productivity; and iii) educational environments should be designed to facilitate cognitive performance (e.g., attention, memory, and comprehension), and creating motivating and inspirational spaces alongside reducing stress and providing refuge/ feelings of safety and security for the student populations – as such mapping onto the four theories of biophilic benefit. Therefore, this study examines the impact of biophilic features in standard educational spaces (i.e., classrooms, corridors, and stair wells). We also examined whether emotional responses to biophilic quality vary as a function of demographic information (i.e., age, sex, ethnicity, and environment conditions they grew up and were currently living in). We hypothesised that the greater the biophilic quality, the more positive emotional impacts will be seen in terms of *Stress Recovery, Attention Restoration, Refuge and Prospect, and Inspiration.*

## 2. Methodology

### 2.1 Participants and ethical statement

Participants (n = 255) were recruited from university staff, students, and the general population and via a research platform Prolific with a network of over 100k active participants [28]. The survey was conducted between 26/04/2022 and 18/05/2022 as a part of a larger online data collection (total n = 329; not all were exposed to the virtual spaces) using the online survey tools Qualtrics [29] and Gorilla Experiment Builder [30]. The majority of participants were recruited through Prolific from the general population with 63 of the participants recruited from the university staff and student population. Written consent forms for each participant were obtained and stored anonymously in Qualtrics.

Demographic information (i.e., age, biological sex, ethnicity and environmental conditions participants grew up and were currently living in during term time and outside termtime) are presented in Table 1. The participants’ ages ranged from 18 to 77 years old (mean age =  39.22; SD =  13.35), and the majority of the participants were females (67%), and of white ethnicity (82%). The characteristics of the living environments of participants (growing up, termtime, and outside termtime) showed good variation from urban to nature (the results of impacts of demographic characteristics will be presented in the results section). Nottingham Trent University Ethics Committee for Non-invasive Research provided a positive opinion of the current study.

**Table 1:** Demographics information of the participants.

Characteristic	N = 255 Sample exposed to the biophilic spaces	
Age Range	18–77 Years	
Biological Sex	Female	67%
	Male	22%
	Withheld	1%
Ethnicity	White	82%
	Black/African American	5%
	Asian	8%
	Other/ declined	4%
Environment Growing Up	Much More Nature	18%
	A Little More Nature	20%
	Equal Nature and Urban	18%
	A little More Urban	18%
	Much More Urban	17%
Living Environment in Termtime	Much More Nature	13%
	A Little More Nature	21%
	Equal Nature and Urban	18%
	A little More Urban	22%
	Much More Urban	17%
Living Environment Outside of Termtime	Much More Nature	16%
	A Little More Nature	19%
	Equal Nature and Urban	21%
	A little More Urban	19%
	Much More Urban	17%

### 2.2 Design & Procedure

**Fig 1:**
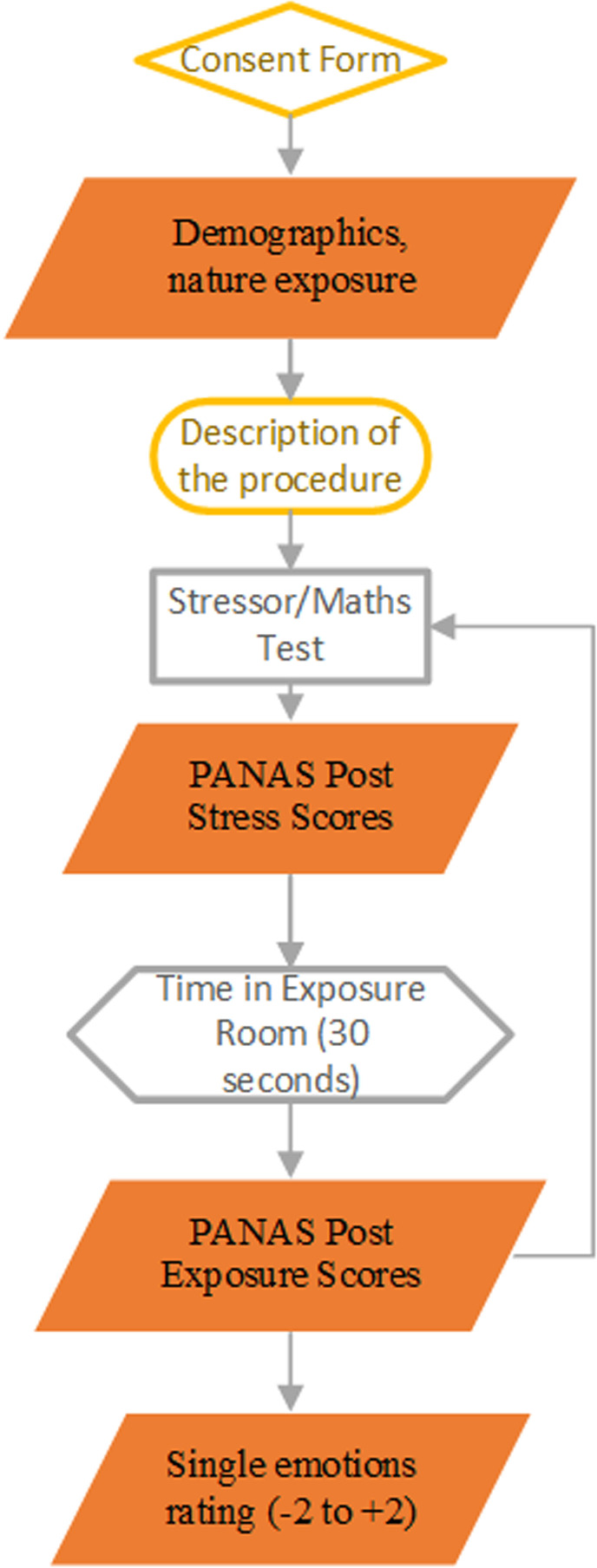
Experimental Procedure.

Using a cross-sectional, within-subjects design, participants rated 2D images of building interiors taken from each of 12 Virtual Reality environments (described in the “The Stimulus at four Biophilic Levels”;as in Fig 1) and presented online using Gorilla (Gorilla, 2016). Prior to each set of images, participants completed a stress-induction task that combined a speeded mental arithmetic test, with unpleasant noise and social stress. During the stress-induction task, participants had twenty seconds to answer as many arithmetic questions as they could. The questions were simple arithmetic operations such as 373 +  386 =  759 pulled from a randomised self-generated database of 240 equations (comprising sixty addition, sixty subtraction, sixty multiplication and sixty division questions). Whilst completing the maths test, participants were exposed to stressful background noises of traffic and construction sounds. Participants were asked to set the volume at a comfortable but audible level in the introduction. Post exposure, they were given the option to declare if they had heard no sound due to technical issues or did not have access to speakers/headphones. To add a social stress element to the task, participants were informed they were being compared to a group of people who had already completed the task, and no matter how well they did, they were always told that they performed more poorly than the comparison group. The task therefore presented participants with three stressors, mental arithmetic, auditory unpleasant noise, and negative comparison against other people.

After the stressor task, participants rated their emotional states using items from the positive and negative affect scale (PANAS) [31]They were then presented with one of four types of recovery stimuli, which systematically varied the level of biophilic design features (Fig 1). These ranged from either no biophilic features to an accumulating set of one, two or three types of biophilic features, in order to examine the impact of increasing levels of biophilic features on state responses. Each of the three images of a specific level were shown for 10 seconds resulting in a total of 30 seconds image display. After each recovery stimulus, participants were asked to give a single rating on whether they liked the designs (ranging from negative =  ‒2 to positive =  + 2). After 30 seconds of viewing the biophilic recovery stimuli, participants rated their emotional states again using PANAS followed by the next stressor task, stimuli display and ratings.

The Positive and Negative Affect Schedule (PANAS) [31] can provide a reliable and independent measures of positive and negative affect, which are useful beyond traditional symptom questionnaires. It was chosen for this study for several reasons. Firstly, it is supported by extensive research and has excellent psychometric properties, high internal consistency Cronbach’s alpha values typically above 0.84 for both the Positive Affect (PA) and Negative Affect (NA) scales has been validated across various populations and languages, showing strong construct validity [31,32] and good convergent validity, correlating well with other measures of similar constructs, and good discriminant validity, distinguishing between different constructs [33]. Furthermore, it is brief and simple to administer, making it ideal as a part of a wider battery of psychometric questions. Finally, the PANAS has been used in a wide range of studies, including those focusing on mindfulness, emotional disorders, and general wellbeing. Its adaptability to different contexts and populations enhances its utility [32,33] and this in particular was of interest to us in using it to mirror that kind of affect elicited by each of the biophilic theories.

### 2.3 The Stimulus at four Biophilic Levels

**Fig 2:**
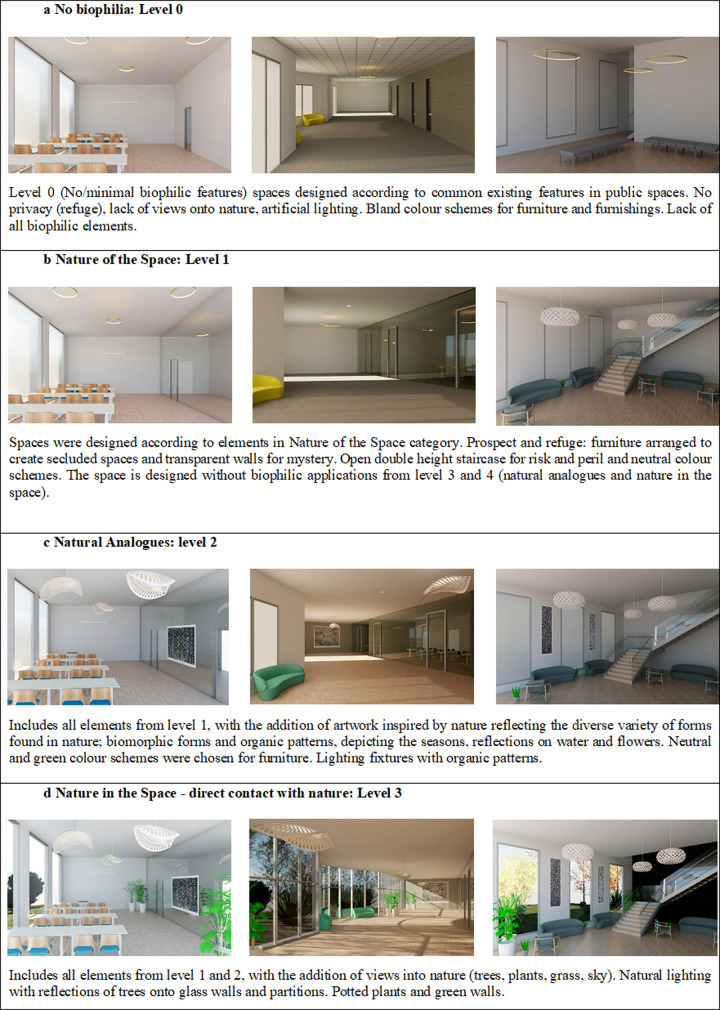
Overview of stimuli created across four levels of biophilic design.

Three types of spaces are explored, namely, the classroom, corridors and staircases. According to leading biophilic framework [34], three biophilic features (i.e., nature of the space, natural analogues and nature in spaces) are incrementally added to the spaces. In total, 12 scenes (stimuli) of three public spaces were manipulated across four different levels (no biophilic features to one, two or three types of biophilic features) were created using Autodesk Revit rendering tools (as shown in Fig 2). The biophilic levels reflect the three categories (i.e., nature of space, natural analogue and nature in spaces) based on theoretical frameworks on the application of biophilic design (Browning 2020). More detailed descriptions for each level of the stimulus can be found in Fig 2.

### 2.4 Self-reported Response PANAS Scores

Eight items from the Positive and Negative Affect Scale (PANAS) [35] mapping onto the four proposed theories (i.e., recovery, attention, and refuge, Inspiration) were used to assess the psychological responses to stress-induction and stimuli displays (see Table 2). One positive and one negative state-word were used for each construct, respectively. PANAS is a self-report questionnaire that asks the participant to rate how they are feeling “right now” across adjectives of positive and negative affect. Items were rated on a 5-point scale ranging from 1 (not at all) to 5 (very much). PANAS assessments of positive and negative affect have shown excellent reliabilities (Cronbach alpha = .910; [36]. After reverse-scoring of negative mood items, ratings for the two relevant items were summed. Biophilic scores (B_F_) for each of the four theories were calculated by subtracting the base score (i.e., post-stress induction scores B_O_) from score following each 30 second recovery period (B_x_) to indicate mood change:

**Table 2:** Relationship between Biophilic Theoretical Constructs and PANAS Scores.

Biophilia Theory	PANAS Items
Stress Reduction	Relaxed, Irritable
Attention Restoration	Attentive, Fatigued
Refuge and prospect	Self-assured, Frightened
Inspiration	Inspired, Downhearted

1) *B*_*O*_ *=  PANAS positive item +  PANAS negative item (reverse scored) at post stress stage*
2) *Bx =  PANAS positive item +  PANAS negative item (reverse scored) at Post image exposure*
3) *B*_*F*_ *=  B*_*x*_ *- B*_*O*_

Thus, the higher the B_F_ score, the greater the positive mood change from stress induction stage to post-recovery stage.

### 2.5 Statistical Analyses

Data were analysed and cleaned using IBM SPSS 26.0. PANAS item scores were summed, missing scores were replaced with a mean score for no more than two entries per participant. Data were assessed for skew and kurtosis, which fell within the range for normal distribution. Zero-order correlations assessed associations between demographics and biophilic mood ratings. Analyses of Variance (ANOVA) were used to test for the effect of recovery level (0, 1, 2, 3) on *B*_*F*_. Separate ANOVAs were conducted for each of the biophilic constructs (Recovery, Attention Restoration, Refuge, Inspiration). *Post-hoc* tests explored differences between specific biophilic levels.

## 3. Results

### 3.1 Demographics

Pearson Correlations were weak and non-significant between PANAS-derived biophilic theoretical construct scores (see 2.4) and demographic variables indicating that biophilic manipulations affected all participants regardless of different age, biologic sex, ethnicity groups, and nature exposure (including growing up environments and current living environments).

### 3.2 Differences Between Biophilic levels for Each of the Four Biophilic Theories

The ANOVA results revealed significant effects of biophilic level on mean ratings of Restoration, Attention Recovery, Refuge and Inspiration (see Table 3) showing that the biophilic levels significantly affected the PANAS derived mood change scores mapping onto the four biophilic theoretical constructs. As shown in Table 4, the Biophilic mean scores gradually increased with each level. Scores for Level 0 (no biophilic features) were negative, ranging from ‒0.84 for inspiration to ‒0.37 for recovery (indicating reductions in positive affect), whereas the positive scores were seen for all three Biophilic levels – the highest for Level 3 (most biophilic features), ranging from 0.7 for attention restoration to 1.74 for recovery.

**Table 3:** Biophilic Theoretical Constructs ANNOVA results. Descriptive Statistics and ANOVA Results of the Biophilic Constructs Across Biophilic Levels.

*Biophilic Level N = 255*	*0*	*1*	*2*	*3*	*F* *(df1, df2)*	*p*	η2
**Recovery**							
**Mean** **(SD)**	‒.37 (1.63)	.85 (1.76)	1.13 (1.61)	1.74 (1.87)	105.38 (2.67, 741.91)^1^	<.001	.27
**Attention**							
**Mean** **(SD)**	‒.36 (1.29)	.036 (.82)	.32 (1.17)	.70 (1.25)	45.44 (2.46, 268.53)^1^	<.001	.14
**Refuge**							
**Mean** **(SD)**	‒.28 (1.52)	.032 (.05)	.32 (1.09)	.51 (1.17)	27.03 (2.29, 635.41)^1^	<.001	.09
**Inspiration**							
**Mean** **(SD)**	‒.84 (1.63)	.44 (1.44)	.62 (1.37)	1.19 (1.71)	105.38 (2.46, 582.80)^1^	<.001	.28

^1^Mauchley’s test was found to be significant across measures therefore, degrees of freedom were corrected using Huynh-Feldt estimates of sphericity.

**Table 4:** Bonferroni Post-hoc Analyse of Biophilic Constructs across Biophilic Levels.

Biophilic Construct	Significant Differences between Biophilic Levels	Non Significant Differences
Stress Recovery	0 < 1***, 2***, 3***; 1 < 3***; 2 < 3***	1 < 2
Attention Restoration	0 < 1***, 2***, 3***; 1 < 2*; 2 < 3***	1 < 3
Refuge	0 < 1***, 2***, 3***; 2 < 3*	1 < 2,3
Inspiration	0 < 1***, 2***, 3***; 1 < 3***; 2 < 3***	1 < 2

Note: significance levels at

***p < 0.001,

**p < 0.005,

*  p < 0.5

**Fig 3:**
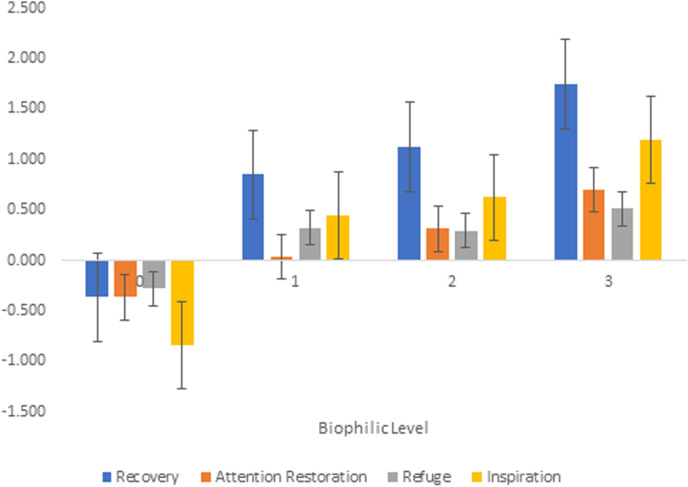
Estimated Marginal means Across Biophilic levels.

**Fig 4:**
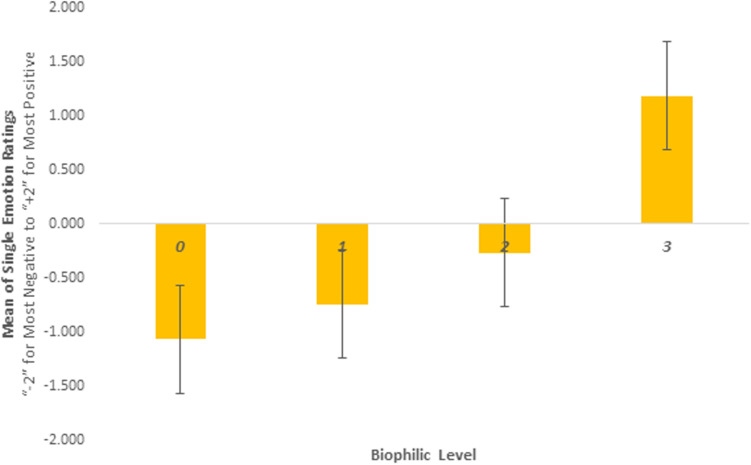
Single Emotion Rating).

Post-hoc tests revealed significant differences between most biophilic levels as shown in Table 4. Biophilic change scores for Recovery, Attention, Refuge and Inspiration were significantly lower for level 0 compared to levels 1, 2 and 3, and consistently higher for level 3 compared to level 2 (and level 1 though only for Recovery and Inspiration). Attention Restoration change scores were also significantly higher for level 2 compared to level 1. These results are represented graphically in Fig. 3 and Fig. 4.

### 3.3 Single rating of the designs

Fig 4 shows mean ratings for the single item question of liking the stimuli. It is interesting to see that all the means for level 0 to level 2 are negative, only level 3 is positive.

## 4. Discussion and Conclusion

In this study, a novel interdisciplinary research methodology framework was developed to assess the effects of the built environment on emotional states. This framework incorporates biophilic designs and psychological research methods. The emotional impacts were systematically examined based on three existing biophilic theories (attention, stress recovery, refuge) and one new biophilic hypothesis—biophilic inspiration. Utilizing a cross-sectional, within-subjects design, the study engaged 255 participants to evaluate 2D images of building interiors across these levels. The Positive and Negative Affect Scale (PANAS) was used to measure participants’ emotional responses before and after biophilic stimuli exposure, following a stress-induction task. The findings showed the significant roles of the built environment in promoting wellbeing and stimulating inspiration. Additionally, it was observed that the emotional responses did not show significant variations based on demographic factors such as age, sex, or the nature of the participants’ past or present living environments. This may indicate broader positive impacts on wellbeing, regardless of the broad demographic variations examined in this study. This research offers fresh empirical evidence that deepens our understanding of the relationship between biophilic design elements and their motivational and inspirational effects. This study not only broadens the theoretical foundations of biophilic design but also underscores the necessity for ongoing research into how these design principles can be effectively applied across various architectural settings to promote human flourishing.

This paper is the first to explore the biophilic inspiration hypothesis. The method developed in this study is a unique interdisciplinary approach to investigating a novel biophilic inspiration theory, which is supported by the experimental findings. Inspiration remains a relatively underexplored domain at the intersection of environmental psychology and architecture. but crucial for creating spaces that encourage creativity, enhance wellbeing, and boost productivity. This research is particularly pertinent in the design of educational (e.g., university buildings) and occupational (e.g., workplace) environments, where the surrounding atmosphere is pivotal in influencing the experiences and outcomes of its occupants.

Existing biophilic design research often oversimplifies or overlooks the complexity of developing biophilic features, and the impacts of various levels of biophilic intensity are rarely studied. In this paper, a novel method of incremental variation of the biophilic levels was created based on theoretical frameworks for applying biophilic design principles [3]. The biophilic features were organised into four levels (No biophilia: Level 0; Nature of the Space: Level 1; Natural Analogues: level 2; Nature in the Space - direct contact with nature and encompasses a full integration of natural features, analogues, and direct nature contact Level 3). The research results presented in this paper expand our understanding of healthy buildings through biophilic design principles.

Firstly, the study provides evidence of discernible negative emotional reactions to the absence of biophilic features (i.e., Level 0). The negative psychological responses to non-existent or insignificant biophilic designs can potentially explain the phenomenon known as ‘sick-building syndrome’. The term “sick building syndrome” (SBS) typically describes situations in which building occupants experience acute health and comfort issues that are linked to time spent in a building [4]. Currently, sick building research mostly focuses on poor indoor air quality and inadequate ventilation [37,38], which are crucial, but overlooked psychological wellbeing. One significant contribution of this study is that it illuminates potential underlying psychological causes of “sick building syndrome” in non-biophilic buildings, which are common practices worldwide [5,27].

Secondly, incremental improvements were observed when participants were exposed to progressively enhanced biophilic designs (from Level 1 to Level 3). These enhancements led to significant changes in stress reduction and recovery-related responses (e.g., feeling more relaxed), attention restoration (e.g., being less fatigued), feelings of refuge and prospect (e.g., feeling safe), and increased inspiration (e.g., feeling inspired and upbeat). Rapid urbanization separates us from our natural environment. Furthermore, as a species, we are becoming more stressed and less connected to nature (which itself is under threat) [2]. This study substantiates the role of biophilic design in mitigating the effects of urbanisation and associated sick building syndrome by examining various biophilic elements. Non-biophilic environment worsened the affective states, whereas the enhanced biophilic environment improved it gradually in terms of perceived stress reduction, regaining attentional resources, feelings of safety and inspiration. It advocates for the incorporation of multifaceted biophilic design features—such as nature in space, nature analogues, and the nature of spaces into architectural practices. This research also calls for interdisciplinary research to integrate diverse biophilic features into architectural practices systematically.

The research highlights the significant role that biophilic design research can play in studying the psychological impacts of the built environment. However, there are some limitations of this study which require further research. In this study, we included 63 participants (university staff and students) and 192 participants (general population through Prolific), totalling 255 participants. The acceptable sample size was determined based on the rule of thumb for quantitative analysis [39,40]. Future studies will aim to include a larger sample size. Additionally, a follow-up manuscript is planned, focusing on personality trait data collected from this cohort and employing structural equation modelling. In this paper, biophilic designs were systematically varied and organised, albeit limited to only four levels. Biophilic design is a complex endeavour. There is a lack of metrics for categorising biophilic designs. Future research is needed to develop more finely categorised and personalised biophilic features. While the current study focused on visual stimuli, the investigation of other sensory modalities (e.g., biophilic smell, touch, sound) should also be developed. This study was well-controlled using carefully designed digital environments allowing the systematic manipulation of different biophilic design features. Future studies need to validate the findings in truly naturalistic settings (e.g., in-site in a built environment, in a park or urban forest). In this paper, limited demographics (i.e., age, gender, and places of living) were considered. Future research is needed to explore more fine-grained individual differences (e.g., personality traits, neurodiversity, and sensory sensitivities). This paper utilised subjective self-reporting methods (i.e., PANAS rating), future research can be developed to use more objective measures such as different biophysiological wearable sensors. Advances in wearable technology, affordably, offer the potential to gather reliable physiological data in real-time, enabling more comprehensive assessments of the impacts of biophilic design on psychological states.

Throughout the last century, architectural design has largely concentrated on structural breakthroughs, notably in the construction of high-rise buildings, or on enhancing energy efficiency. Yet, these advances have frequently neglected the vital influence that nature connection and architectural shapes and spaces have on our emotional health. Under the *positive psychology* lens [41], the empirical findings presented herein underscore the critical importance of biophilic design not only in addressing sick building phenomena but also in fostering healthier and flourishing environments within the built landscape. Biophilic design is interdisciplinary in nature and requires effective communication and sustained interdisciplinary interactions. Innovative collaboration among end users, architects, designers, engineers, and psychologists is essential for identifying and integrating these features into biophilic solutions enhancing both human wellbeing and planetary health.
